# Anisotropic light propagation in human brain white matter

**DOI:** 10.1117/1.NPh.12.4.045003

**Published:** 2025-10-07

**Authors:** Ernesto Pini, Danila Di Meo, Irene Costantini, Michele Sorelli, Samuel Bradley, Diederik S. Wiersma, Francesco S. Pavone, Lorenzo Pattelli

**Affiliations:** a Istituto Nazionale di Ricerca Metrologica, Turin, Italy; b European Laboratory for Non-linear Spectroscopy, Sesto Fiorentino, Italy; c University of Florence, Department of Biology, Sesto Fiorentino, Italy; d University of Florence, Department of Physics and Astronomy, Sesto Fiorentino, Italy; e National Research Council – National Institute of Optics, Sesto Fiorentino, Italy

**Keywords:** multiple scattering, time-resolved measurements, anisotropic diffusion, scattering tensor, neurophotonics, Monte Carlo simulations

## Abstract

**Significance:**

Accurate modeling of light diffusion in the human brain is crucial for applications in optogenetics and spectroscopic diagnostic techniques. White matter tissue is composed of myelinated axon bundles, suggesting the occurrence of enhanced light diffusion along their local orientation direction, which however has never been characterized experimentally. Existing diffuse optics models assume isotropic properties, limiting their accuracy.

**Aim:**

We aim to characterize the anisotropic scattering properties of human white matter tissue by directly measuring its tensor scattering components along different directions and correlating them with the local axon fiber orientation.

**Approach:**

Using a time- and space-resolved setup, we image the transverse propagation of diffusely reflected light across two perpendicular directions in a post-mortem human brain sample. Local fiber orientation is independently determined using light sheet fluorescence microscopy and two-photon fluorescence microscopy.

**Results:**

The directional dependence of light propagation in organized myelinated axon bundles is characterized via Monte Carlo simulations accounting for a tensor scattering coefficient, revealing a weaker scattering rate parallel to the fiber orientation. The effects of white matter anisotropy are further assessed by simulating a typical time-domain near-infrared spectroscopy measurement in a four-layer human head model.

**Conclusions:**

We provide a first characterization of the anisotropic scattering properties in post-mortem human white matter, highlighting its direct correlation with axon fiber orientation, and opening the way to the realization of quantitatively accurate anisotropy-aware human head 3D meshes for diffuse optics applications.

## 1 Introduction

The intricate macroscopic and microscopic structure of the human brain poses a significant challenge for techniques that rely on diffuse near-infrared light for functional imaging and diagnostics.^1^ The complex and heterogeneous composition of brain tissue influences light propagation, affecting the accuracy of these techniques. Current optical methods, such as functional near-infrared spectroscopy (fNIRS) and diffuse optical tomography (DOT), focus primarily on monitoring hemodynamic responses by mapping blood oxygenation changes in different brain regions.^2^^–^^5^ Among these, time-resolved techniques play a crucial role in extracting absolute tissue oxygen saturation values and estimating the reduced scattering coefficient,^6^ both of which are essential for functional and clinical assessments. A key advantage of these methods is their ability to separate extra-cerebral and cerebral contributions, as different photon propagation times correspond to different penetration depths. This feature enhances the accuracy of functional imaging and disease diagnostics by isolating signals from deeper brain structures, as long as light transport through brain tissue is accurately modeled.

In addition to neuroimaging, light diffusion in brain tissue is also relevant to optogenetics, a technique enabling precise, cell-specific control of neural circuits using light-sensitive proteins. Optogenetics allows for stimulating or inhibiting neurons with high temporal and spatial precision,^7^^–^^9^ which however requires accurate modeling of light transport in neural tissue to ensure that light delivery reaches the desired depth and intensity for activating targeted cells. The high scattering of brain tissue significantly influences the illumination profile, affecting the efficiency of optogenetic activation, especially for deep-brain stimulation via optical fibers. Although previous studies examined how light propagates through brain tissue,^10^ isotropic properties are often assumed in spite of the apparent structural anisotropy of white matter tracts. Similarly, more realistic modeling of light diffusion in the brain could benefit transcranial photobiomodulation techniques, which rely on localized scalp illumination to stimulate brain function and potentially treat neurological and psychiatric disorders.^11^^–^^13^

White matter is composed of myelinated axons that are locally organized to form bundles. The preferential alignment of these bundles creates a directional dependence in light diffusion, as elongated structures interact differently with light depending on its propagation direction. This anisotropic behavior has been well-characterized in diffusion-weighted magnetic resonance imaging (DW-MRI) and diffusion tensor imaging (DTI), where axonal structures are mapped based on water diffusion patterns.^14^ However, anisotropy in diffuse optics remains largely disregarded, despite its potential impact on optical neuroimaging and optogenetics, with a lack of experimental and numerical validation for anisotropic scattering coefficients in human white matter.

A major challenge in modeling brain tissue optics is the scarcity of widely accepted reference values for optical properties, which complicates the development of standardized models. Experimental studies report highly variable results,^15^^–^^20^ often due to differences in sample preparation (e.g., formalin fixation, paraffin embedding)^21^ or measurement conditions such as temperature-dependent scattering changes.^22^^,^^23^

In this context, studies in the field of diffuse optics addressing the structural anisotropy of white matter are even scarcer. Hebeda et al.^24^ reported a first measurement of the effective attenuation coefficient $\mu_{\mathrm{eff}}$ along parallel and perpendicular directions with respect to white matter axons in bovine brain, finding significant directional differences: $\mu_{\mathrm{eff},∥}=(0.47\pm 0.06)\;{\mathrm{mm}}^{-1}$, $\mu_{\mathrm{eff},⊥}=(0.63\pm 0.13)\;{\mathrm{mm}}^{-1}$ at 633 nm. Heiskala et al.^25^^,^^26^ attempted a description of anisotropic light transport in the whole human head using a simplistic version of the anisotropic diffusive equation and Monte Carlo (MC) simulations using parameters derived from DTI measurements as a proxy for light diffusion anisotropy. Although this approach allowed for the recognition of the presence of transport anisotropy in white matter, it failed to accurately predict its effects in single-frequency-domain DOT measurements. In addition, no attempt was made to measure the actual direction-dependent scattering coefficients, which were simply assumed to be proportional to the DTI fractional anisotropy data for water diffusion through white matter tissue. More recently, De Paoli et al.^27^ measured direction-dependent reduced scattering coefficients in spinal cord white matter, reporting a large transport anisotropy associated with the high degree of alignment of axons in the spinal cord. Relevant works in this direction have also been published by Menzel et al.,^28^^,^^29^ who modeled the anisotropic geometry of individual nerve fiber bundles using finite-difference time-domain simulations, and showed that the angular distribution of light scattered from ultrathin brain slices depends on the local axon orientation. These results are also of high interest to understand whether myelinated axon fibers can favor the directional propagation of biophotons in brain tissue, a topic which received considerable attention in recent years.^30^^,^^31^

Despite these efforts, an accurate experimental characterization of the direction-dependent scattering coefficient inside human brain white matter is still lacking. In this work, we introduce a validated tensor-based model for the description of anisotropic light transport, enabling us to overcome previous oversimplifications of scattering in structurally anisotropic media. Our experimental characterization spans time scales covering both diffusive propagation regimes and sub-diffusive transients, providing new insights into the optical properties of white matter, with implications for neuroimaging, optogenetics, and computational light transport models.

## 2 Anisotropic Diffusion Model

In anisotropic materials, the diffusive constant D and the scattering coefficient $\mu_{s}$ can be represented as 3×3 tensor quantities D, $\mu_{s}$ which can usually be diagonalized using an appropriate common reference frame. In analogy to the isotropic case, the diffusion tensor elements can be associated with reduced scattering coefficients along different directions $\left(\mu_{s,x}^{\prime },\mu_{s,y}^{\prime },\mu_{s,z}^{\prime }\right)$

$$D=(\begin{array}{ccc} D_{x} & 0 & 0\\ 0 & D_{y} & 0\\ 0 & 0 & D_{z} \end{array})=(\begin{array}{ccc} \frac{1}{3}\frac{v}{\mu_{s,x}^{\prime }} & 0 & 0\\ 0 & \frac{1}{3}\frac{v}{\mu_{s,y}^{\prime }} & 0\\ 0 & 0 & \frac{1}{3}\frac{v}{\mu_{s,z}^{\prime }} \end{array}),$$ (1)

having dimensions of $\left[m^{2}\;s^{-1}\right]$, where $v=c/n_{\mathrm{eff}}$ represents the speed of light in the medium, with c as the speed of light in vacuum and $n_{\mathrm{eff}}$ as the medium’s effective refractive index.

On the contrary, at the microscopic level, we can define a diagonal scattering coefficient tensor

$$\mu_{s}=(\begin{array}{ccc} \mu_{s,x} & 0 & 0\\ 0 & \mu_{s,y} & 0\\ 0 & 0 & \mu_{s,z} \end{array}).$$ (2)

In the diffusive regime, analytical relations between these two tensors have been recently derived for the case of uniaxial structural asymmetry^32^ typical of aligned fiber bundles, revealing a non-trivial dependence between each component of the diffusion tensor and the microscopic scattering coefficients along all three directions. Compared with the simplistic assumption that each component of the diffusion tensor depends exclusively on its corresponding scattering coefficient, the exact solution allows for avoiding systematic errors, the magnitude of which is directly related to the degree of structural anisotropy.^33^

In general, before the onset of diffusion, scattering asymmetry is also expected to influence light transport, as often modeled through the Henyey-Greenstein phase function with an asymmetry factor g. In analogy with μ and D, the asymmetry factor itself could take a tensor form in anisotropic media, which however would double the number of free parameters and make the quantitative retrieval of optical properties practically unfeasible. In diffusive isotropic media, this is typically not a problem thanks to the existence of a simple “similarity relation” linking the scattering coefficient to its reduced counterpart $\mu_{s}^{\prime }=\mu_{s}(1-g)$. However, this simple relation does not hold in the anisotropic case, and the microscopic interpretation of the reduced scattering coefficient in the random walk picture of diffusion is lost. To restore a microscopic quantity of practical utility, we introduce a trade-off by considering a tensor scattering coefficient and a scalar asymmetry factor, which we combine into an *effective reduced scattering coefficient* ${\overset{˜}{\mu }}_{s,i}^{\prime }$^34^^,^^35^

$${\overset{˜}{\mu }}_{s,i}^{\prime }=\mu_{s,i}(1-g)\neq \mu_{s,i}^{\prime },$$ (3)

with i={x,y,z}. The effective reduced scattering coefficient accounts for the directional dependence of scattering in anisotropic media, providing a more accurate description of light transport especially in the case of biological tissues, where the asymmetry factor is expected to be close to unity irrespective of the direction.

The main aim of this study is to quantitatively determine the components of the ${\overset{˜}{\mu }}_{s,i}^{\prime }$ tensor and of the g scalar by looking at light transport dynamics during the early transient and the subsequent diffusive regime.

To quantify the degree of light diffusion anisotropy, we further introduce an *Optical Fractional Anisotropy* (OFA), inspired by the concept of fractional anisotropy (FA) commonly used in the field of DTI^36^ (based on water molecule diffusion) or in structure tensor (ST) analysis^37^ (based on histological sections microscopy). The OFA follows the same definition, using the components of the light diffusion tensor

$$\mathrm{OFA}=\sqrt{\frac{1}{2}\frac{(D_{x}-D_{y}{)}^{2}+(D_{y}-D_{z}{)}^{2}+(D_{z}-D_{x}{)}^{2}}{D_{x}^{2}+D_{y}^{2}+D_{z}^{2}}},$$ (4)

and is similarly comprised between 0 and 1 for isotropic diffusion and perfectly directional propagation, respectively.

## 3 Materials and Methods

### 3.1 Sample Preparation

Human tissue samples were procured through the body donation program (Association des dons du corps) of the Université de Tours. Written consent was obtained from healthy participants prior to death, including the brain for any educational or research purposes. The authorization documents of the Association des dons du corps are kept with the Body Donation Program at the Université de Tours and were collected within the general frame of the approved IRB submission to the Partners Institutional Biosafety Committee (PIBC, protocol 2003P001937). Upon collection, samples were placed in neutral buffered formalin (pH 7.2 to 7.4) and stored at room temperature. Blocks from the fixed samples were washed with phosphate-buffered saline solution at 4°C with gentle shaking for 1 month.

### 3.2 Optical Gating Setup

**Fig. 1:**
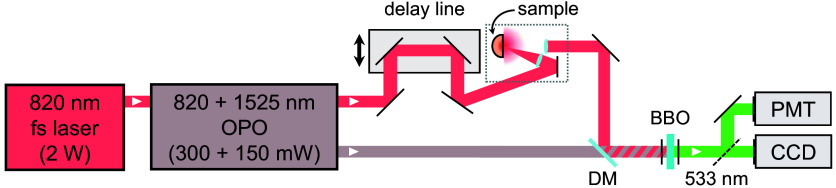
Sketch of the experimental setup for time- and space-resolved measurements when using an 820-nm probing wavelength. A titanium-sapphire fs laser is used to pump an optical parametric oscillator (OPO). The probe and gate beams are recombined using a dichroic mirror (DM) onto a β-barium borate (BBO) nonlinear crystal, generating a sum-frequency signal that is detected with either a charge-coupled device (CCD) camera or a photomultiplier tube (PMT). The probe pulse impinges on the sample at an off-axis angle of θ=13  deg, whereas light is collected perpendicularly to the sample surface, thus excluding light that is specularly reflected at the sample interface.

Our study considered diffuse reflectance from a piece of post-mortem human brain pericalcarine cortex with dimensions of roughly 18  mm×15  mm×20  mm, presenting both gray and white matter regions. Utilizing near-infrared light at λ=820  nm, we performed time- and space-resolved reflection measurements on the white matter region due to its known fibrous structure composed of locally aligned bundles of neuron axons. Anisotropic light transport was studied by means of a transient imaging apparatus based on an optical gating scheme (Fig. 1), allowing the capture of the spatio-temporal evolution of reflected intensity with sub-ps resolution upon illumination with a focused pulse with a duration of ∼150  fs.^35^^,^^38^ The laser sources emit trains of synchronous pump-gate pulses at different wavelengths, which are recombined before detection in a β-barium borate (BBO) nonlinear crystal, where a sum-frequency conversion occurs producing a signal at a visible wavelength. The gate beam is expanded to achieve a flat intensity distribution over the nonlinear crystal surface to obtain a uniform upconversion efficiency of the signal over the whole field of view. A motorized delay stage is used to adjust the relative delay between the probe and gate arms of the setup, thus allowing the resolution of the fast dynamics of light diffusion.

A series of transient frames was recorded with a CCD camera at different times between 0.5 and 15.5 ps with 1 ps incremental steps. The measurements were taken over a circular field of view with an area of $13.2\;{\mathrm{mm}}^{2}$, averaging over different adjacent positions across a $∼1\;{\mathrm{mm}}^{2}$ white matter region to reduce noise, cover a more representative sample area, and average over speckle features to obtain an incoherent intensity profile. Within this limited time window encompassing just a few ps, the sample can be effectively considered homogeneous and semi-infinite because the observed diffused patterns remain bound within the white matter region both in the transverse plane and along the depth direction.

Notably, the functional shape of the measured profiles is independent of absorption, as the presence of absorption results in a mere modulation of their amplitude, thus allowing for the retrieval of scattering parameters unaffected by confounding cross-talk effects. Quantitative information on the absorption coefficient can be retrieved by looking at the integrated time-resolved intensity instead, which can be obtained either by directly accumulating the intensity in each pixel or using a photo-multiplier tube as a detector for a more convenient sampling along the time axis.

### 3.3 Fiber Orientation Analysis

**Fig. 2:**
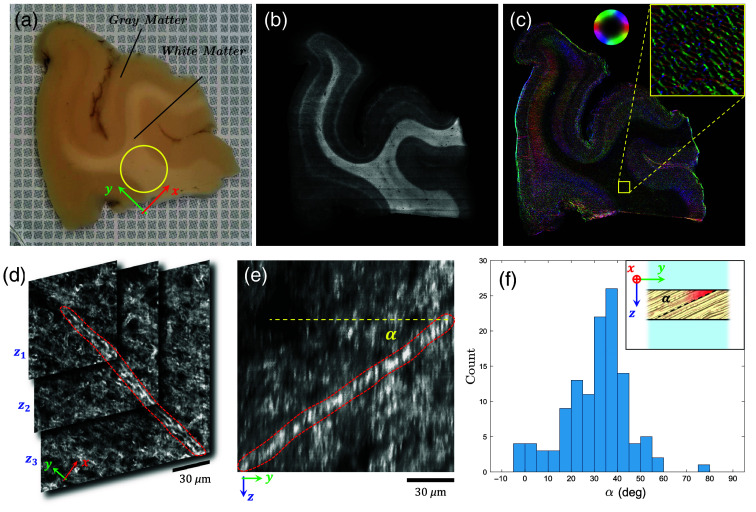
(a) 1-mm-thick brain slice placed on millimeter grid paper for reference. The yellow circle represents the circular field of view selected for the transient imaging measurements. (b) Raw acquisition image obtained with light-sheet fluorescence microscopy after clearing and (c) corresponding axon fiber orientation map. The color wheel indicates the orientation angle within the surface plane. The yellow square marks the $1.1\times 1.1\;{\mathrm{mm}}^{2}$ region of interest for the orientation analysis. Horizontal red features visible across the image are due to striping artifacts in the LSFM measurement. (d) Example of a stack of three TPFM frames in the xy plane at different depths $z_{i}$ (grayscale levels represent the fluorescent intensity). The dashed red curve highlights a fiber (appearing as two parallel lines corresponding to the myelin sheath tube section) coming progressively into view when scanning the acquisition stack along z (Video 1, MP4, 43.1 MB [URL: https://doi.org/10.1117/1.NPh.12.4.045003.s1]). (e) Illustrative fiber view obtained by a dynamic reslicing of the image stack along the yz plane, showing clearly its elevation angle α. (f) Distribution of elevation angles for an ensemble of 120 fibers. A schematic depiction of the reference frame is shown in the inset.

To correlate the anisotropic diffusion and scattering tensor components with the structural anisotropy at the same sample location, we performed an orientation analysis using a custom-made light-sheet fluorescence microscopy (LSFM) setup.^39^ In detail, a 1-mm-thick slice was cut from the sample surface [Fig. 2(a)] with a vibratome (Leica VT1000 S) and subsequently processed using the SHORT-DiD protocol^40^ to achieve tissue optical clearing and specific labeling of myelinated fibers. The sample was imaged via LSFM with an isotropic resolution of 3.6  μm [Fig. 2(b)], and the reconstruction was analyzed with the Foa3D tool^41^ producing an in-plane fiber orientation map of the investigated white matter region with μm-scale resolution.

The analysis confirmed that the investigated white matter region is characterized by a homogeneous fiber density with a well-defined local alignment. This allows us to assume a spatially uniform scattering anisotropy tensor and reference frame for the diagonalization of the scattering and diffusion tensors, both aligned along the local axon orientation. Figure 2(c) shows the reconstructed fiber orientation of the whole sample in the sample surface plane, corresponding to the xy plane. To obtain information of the average elevation angle α of the fibers into the sample plane, we further performed two-photon fluorescence microscopy (TPFM) measurements on the same slice [Fig. 2(d)] taking several acquisitions with a voxel side of $0.3\times 0.3\times 1\;\mu m^{3}$ across a region of $1.1\times 1.1\;{\mathrm{mm}}^{2}$. Further technical details on the custom TPFM specifications are provided in a previous publication.^42^ The average elevation angle was finally determined by manually segmenting 120 myelinated fibers randomly selected across the overall investigated area and retrieving their elevation angle via a dynamic reslicing of the image stack (Video 1), as exemplified in Fig. 2(e). This process returned an average elevation angle of α=31  deg±2  deg with a standard deviation of $\sigma_{\alpha }=14\deg$ [Fig. 2(f)]. As a consequence, with respect to the diffusion rates $\left(D_{x},D_{y}\right)$ observed experimentally on the sample surface, the actual anisotropic diffusion tensor elements $\left(D_{⊥},D_{∥}\right)$ parallel and perpendicular to the prevalent orientation direction of the axon bundles will be given by $D_{x}=D_{⊥}$ and $D_{y}=D_{∥}\;\cos(\alpha )$, where we have assumed transport along the two perpendicular axes to be the same, i.e., that the arrangement of axon fibers in the bundle is uniaxially symmetric on average around its main orientation axis.

## 4 Results

### 4.1 Transient Imaging Measurements

**Fig. 3:**
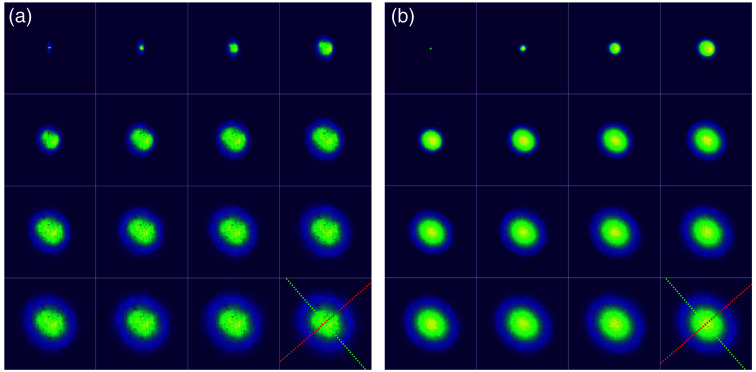
(a) Transient imaging measurements of the reflected intensity distribution, ordered from left to right, top to bottom, with increasing time delay step of 1 ps. Each frame is normalized to its maximum intensity and covers an area of $5.4\times 5.4\;{\mathrm{mm}}^{2}$. (b) Corresponding best-fit Monte Carlo simulation. The red and green dotted lines represent the x,y axes on the sample surface.

**Fig. 4:**
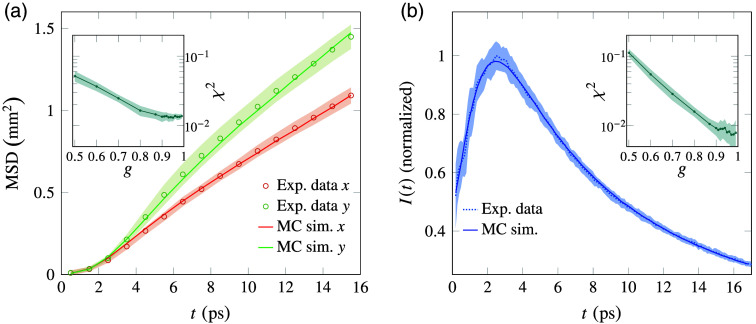
(a) MSD evolution along the x and y directions. (b) Time-resolved reflected intensity. Solid lines represent the best-fit MC simulation. Shaded areas represent (a) the compounded uncertainty from the image acquisition and the bi-variate Gaussian fit and (b) 1σ of the average between five different sample positions. The insets show the fit $\chi^{2}$ dependence on g for the MSD evolution and time-resolved curves, respectively.

Experimental and simulated transient snapshots of the reflected intensity distributions are shown in Fig. 3. The profiles exhibit a faster diffusion rate along the direction parallel to the fibers, showing a clear hallmark of transport anisotropy. The profiles were fitted with a bi-variate Gaussian model to reconstruct the time evolution of their mean square displacement (MSD) growth along the parallel and perpendicular directions in the rotated system of reference [Fig. 4(a)]. After the initial transient, both curves show a linear growth corresponding to the onset of diffusion, with different slopes associated with the different diffusion rates along the two axes. Performing a linear regression on the expansion rates for t>10  ps and correcting for the elevation angle, the components of the diffusive tensor are finally determined as $D_{⊥}=(1.69\pm 0.06)\times 10^{-2}\;{\mathrm{mm}}^{2}\;{\mathrm{ps}}^{-1}$ and $D_{∥}=(2.47\pm 0.16)\times 10^{-2}\;{\mathrm{mm}}^{2}\;{\mathrm{ps}}^{-1}$. Using these values with Eq. (4) allows us to evaluate the OFA of this white matter region, returning OFA=0.23±0.03. Notably, this value is lower than the water molecule diffusion FA usually found from DTI in white matter, which is around 0.3 to 0.4 in pericalcarine cortex white matter.^43^^,^^44^

We fit the experimental data using simulations performed with *PyXOpto*, a general MC software offering an implementation of the anisotropic tensor model.^45^^,^^46^ The effective refractive index of white matter is assumed to be $n_{\mathrm{eff}}=1.39$, according to the literature.^20^^,^^47^ Taking advantage of the exact separation between scattering and absorption offered by the transient imaging data, the fitting procedure is performed in two stages. First, MSD curves are fitted using $\mu_{s,∥}$, $\mu_{s,⊥}$, and g as free parameters. Once these parameters are fixed, absorption is finally determined from the spatially integrated time-domain reflectance curve, which also provides an independent confirmation of the g value obtained from the MSD fit. The simulated results are shown in Fig. 3 next to the experimental frames, exhibiting good agreement as confirmed by the comparison of the MSD curves [Fig. 4(a)].

Although the fit on the MSD linear region depends mostly on the effective reduced scattering coefficients, the early time transient (t<8  ps) carries information on the single scattering properties. The goodness of the fit, expressed through its $\chi^{2}$ value, exhibits a clear dependence on the scattering asymmetry g, with best agreement obtained for g>0.9 as expected for biological tissues. At such large asymmetry values, a small uncertainty on g can translate into a large variation of the retrieved scattering coefficients. In this regime, the effective reduced scattering coefficients represent a more robust set of parameters, returning ${\overset{˜}{\mu }}_{s,⊥}^{\prime }=(4.4\pm 0.3)\;{\mathrm{mm}}^{-1}$ and ${\overset{˜}{\mu }}_{s,∥}^{\prime }=(2.6\pm 0.3)\;{\mathrm{mm}}^{-1}$. It is worth noting that the value of ${\overset{˜}{\mu }}_{s,⊥}$ is consistent with the most comprehensive estimations of the (assumed isotropic) reduced scattering coefficient for white matter reported in the literature, ranging between $\mu_{s}^{\prime }=(4.6\pm 0.4)\;{\mathrm{mm}}^{-1}$^18^ and $\mu_{s}^{\prime }=(4.1\pm 0.5)\;{\mathrm{mm}}^{-1}$^19^ at our test wavelength of λ=820  nm. We ascribe this agreement to the fact that for a uniaxial anisotropic medium, two out of three directions are characterized by the perpendicular scattering properties, which will therefore have a larger weight on the average transport properties. Nonetheless, our results show that scattering is markedly reduced along the prevalent alignment direction of fiber axon bundles, leading to an enhanced diffusion rate parallel to their orientation.

Regarding gray matter, the LSFM orientation analysis in Fig. 2(c) shows its rapidly varying structural anisotropy, which appears visually enhanced due to the much lower density of axon fibers compared with white matter. This lower density facilitates the identification of individual fibers by the Foa3D tool. Unlike white matter, gray matter also includes several other elements such as somata and dendrites, which are neither tubular nor uniformly oriented; these structures are ignored by the Foa3D output and will typically make light scattering more isotropic. For this reason, transient imaging measurements performed over different gray matter regions do not show appreciable levels of anisotropic transport, consistent with the fact that FA reported from DTI studies for cortical gray matter is also typically much lower than that reported for white matter, in the 0.1 to 0.2 range.^43^

### 4.2 Time-Resolved Measurements

Given the retrieved components of the effective scattering mean free path tensor, we can analyze the integrated time-resolved data to determine the absorption coefficient and obtain an independent estimation of the asymmetry factor, which is known to be related to the early-time transient of time-resolved reflectance curves.^48^^,^^49^ The fitting procedure [Fig. 4(b)] returns an absorption coefficient of $\mu_{a}=(0.043\pm 0.017)\;{\mathrm{mm}}^{-1}$. Previously reported values of the absorption coefficient for white matter are typically larger, in the range of $\mu_{a}=0.07-0.09\;{\mathrm{mm}}^{-1}$,^18^^,^^19^ which would cause time-domain curves to fall much more rapidly than what we observe experimentally. This discrepancy could be explained by the fixation of the sample, which in some cases has been shown to slightly reduce absorption,^21^ or by a slight overestimation of absorption in the previous literature. Regarding the scattering asymmetry, the minimum $\chi^{2}$ for this fit is again obtained for g=0.9 to 0.99, with a minimum at g=0.97, in excellent agreement with the independent MSD estimation. Notably, this result is also consistent with previous estimates in porcine and bovine brain,^16^ whereas it suggests that the widely assumed value for human brain white matter (g=0.87)^18^ may be underestimated. The high sensitivity of our time-resolved approach allows us to discriminate between the single scattering effects at short times and the effect of absorption at longer times, allowing simultaneous retrieval of these two parameters.

### 4.3 Estimation of the Impact of White Matter Anisotropy in Human Head in fNIRS Measurements

**Fig. 5:**
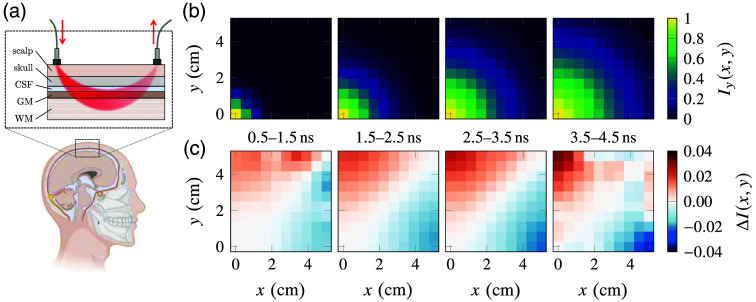
(a) Schematics of the four-layer model used in the MC simulation (created with BioRender). (b) Space-resolved reflectance at different time delays. Light is injected at a position (0, 0) mm and collected over a 10×10 grid with a 50 mm side (representing the upper right quadrant next to the illumination point). The faster diffusion axis of the underlying white matter layer is aligned with the y axis. (c) Relative difference between the space-resolved reflectance ΔI(x,y) of two simulations with orthogonal white matter anisotropy orientation.

For practical functional near-infrared spectroscopy applications, it is interesting to evaluate the potential impact of anisotropic diffusion in the white matter layer. In fact, when using non-invasive techniques, the effect of transport anisotropy in the white matter layer may be partially masked by the presence of the other intervening layers. To this purpose, we compare the results of two MC simulations in a representative four-layer model of the human head, assuming two orthogonal orientations of the white matter layer [Fig. 5(a)]. The four layers represent the scalp/skull, the cerebrospinal fluid (CSF), gray matter (GM), and white matter (WM), respectively. For the first three layers, isotropic scattering and absorption properties are taken from the literature for a wavelength λ=830  nm,^50^ whereas the WM layer is modeled using the experimentally determined parameters and anisotropy. The properties of all layers are summarized in Table 1.

**Table 1:** Properties of the four-layer used for the human head Monte Carlo simulation.^18^^,^^50^^,^^51^ For the white matter, we use the experimentally determined values, replacing $\mu_{s}^{\prime }$ with the direction-dependent effective reduced scattering coefficients $\mu_{s,\|/⊥}(1-g)={\overset{˜}{\mu }}_{s,\|/⊥}^{\prime }$.

	Thickness (mm)	n	g	$\mu_{a}$ (${\mathrm{mm}}^{-1}$)	$\mu_{s}^{\prime }$ (${\mathrm{mm}}^{-1}$)
Skull/scalp	10	1.39	0.89	0.019	0.86
Cerebrospinal fluid	2.5	1.34	0.89	0.0026	0.16
Gray matter	3	1.39	0.9	0.03	0.70
White matter	10	1.39	0.97	0.043	2.6/4.4

In the simulation, the space- and time-resolved reflectance I(x,y,t) is collected from a $100\times 100\;{\mathrm{mm}}^{2}$ region around a central point illuminated with a delta-like pencil beam pulse. This configuration is analogous to a time-domain fNIRS measurement *in vivo* performed with optical fibers placed at different positions from the illumination point, as schematically depicted in Fig. 5(a). Two simulations are performed with the white matter fast-diffusion axis aligned with either the y and x axis, resulting in simulated intensities $I_{y}(x,y,t)$ and $I_{x}(x,y,t)$, respectively. The results of the two simulations, each comprising $10^{11}$ trajectories, are compared by taking their relative difference over time intervals at increasing delays between 0.5 ns and 4.5 ns, by calculating the quantity

$$\Delta I(x,y)=\int_{t_{1}}^{t_{2}}\frac{I_{y}(x,y,t)-I_{x}(x,y,t)}{\frac{1}{2}(I_{y}(x,y,t)+I_{x}(x,y,t))}dt.$$ (5)

The result is shown in Fig. 5(b), where we see that the overall difference increases with the distance and time delay from the illumination point. As expected, the effect of transport anisotropy in the deep WM layer is less pronounced when mediated through the outer head layers. Yet, the overall excess or depletion of light can reach a few percent points along the two directions, even when assuming that all the other layers are isotropic.

## 5 Discussion

This study presents the first quantitative evaluation of the anisotropic scattering tensor components of human brain white matter, linking it directly with its co-located axon orientation. Anisotropic MC simulations are shown to correctly reproduce both the experimental transient intensity profiles and time-resolved reflectance, including very early times associated with single scattering properties such as the asymmetry factor g. Our results show that the (isotropic) scattering coefficient values typically reported in the literature for human white matter are mostly influenced by the scattering coefficient perpendicular to axon fiber bundles, whereas a faster diffusive rate is present in the longitudinal direction.

These findings have implications for neuroimaging techniques such as fNIRS and DOT. Current models assume isotropic scattering; by incorporating anisotropic scattering properties into these models, the accuracy of fNIRS and DOT could be improved, particularly in clinical applications where precise spatial mapping of light scattered through the brain is critical. In the field of optogenetics, accurate modeling of light transport is similarly essential for effective neural stimulation.^10^^,^^52^

Our results suggest that accounting for anisotropic scattering could improve the design of optical stimulation protocols, ensuring that light reaches the intended target neurons with the desired intensity and spatial precision. This could enhance the efficiency of optogenetic activation and photobiomodulation stimulation, particularly for deep-brain regions, where light scattering significantly affects the illumination profile.

The same procedure applied here can be easily implemented to investigate light transport in other structurally anisotropic tissues, such as skin,^53^ muscles,^54^^,^^55^ and tendons,^56^^,^^57^ where directional dependence in light propagation is also typically disregarded when analyzing experimental data, even though it could carry valuable information on the local degree of fiber organization allowing to discriminate between healthy and cancerous tissue.^58^

Also in the human head, additional anisotropy-induced effects may arise when considering more realistic brain models, owing for instance to the fact that other brain tissues besides WM could be structurally anisotropic, or that the outer WM architecture exhibits U-shaped tracts that terminate with a perpendicular orientation to the skull.^14^^,^^59^ With enough sensitivity, these structures might provide a preferential path for probe light to reach a detector inadvertently positioned at the distal end of one of such tracts. Other techniques with enhanced depth sensitivity such as the dual-slope method^60^^,^^61^ could also benefit from a correct accounting of the tensor scattering properties of WM tracts.

In addition, deeper WM regions such as the corpus callosum or the spinal cord are also known to have an even more pronounced structural anisotropy in their axon fiber organization. We expect that these regions would exhibit a higher OFA, leading to a correspondingly larger difference in the directional diffusion rates. Given the large extent of these highly aligned bundles connecting different regions throughout the brain, structural anisotropy may have a major impact on deep brain stimulation^62^ and the probability of detecting diffuse photons even through the whole adult head.^63^ Similar effects could be measured also in infants due to their thinner skull allowing for deeper light penetration into the WM tracts and smaller head size,^64^^,^^65^ even though in this case the OFA may be lower on average as axon fibers myelination progressively increases with age.^66^

In general, the existence of a direct correlation between the local axon bundle arrangement and the orientation of the co-located scattering tensor revealed the presence of a diffusive light transport enhancement along the main alignment direction of the white matter tracts. This result opens the possibility of realizing more accurate and anisotropy-aware 3D meshes of the human head for volumetric light scattering simulations,^50^^,^^52^^,^^67^ building on TPFM 3D orientation analysis^41^^,^^68^ or DTI tractography datasets^14^^,^^59^ as a proxy to the local average orientation of fiber bundles at each location, which could help improve the accuracy of time-resolved transmittance measurements through the human head.^63^^,^^69^

## Supplementary Material

10.1117/1.NPh.12.4.045003.s1

## Data Availability

All the data that support the findings of this study are available from the corresponding authors upon request. Nerve fiber orientation analysis and anisotropic Monte Carlo simulations have been performed using the open-source software tools https://github.com/lens-biophotonics/Foa3D and https://github.com/xopto/pyxopto, respectively.

## References

[1] Ayaz H. et al., “Optical imaging and spectroscopy for the study of the human brain: status report,” Neurophotonics 9(2), S24001 (2022). 10.1117/1.NPh.9.S2.S24001 36052058 PMC9424749

[2] Wheelock M. D. Culver J. P. Eggebrecht A. T., “High-density diffuse optical tomography for imaging human brain function,” Rev. Sci. Instrum. 90(5), 051101 (2019). RSINAK 0034-6748 10.1063/1.5086809 31153254 PMC6533110

[r3] Mahmoodkalayeh S. Ansari M. A. Tuchin V. V., “Head model based on the shape of the subject’s head for optical brain imaging,” Biomed. Opt. Express 10(6), 2795–2808 (2019). BOEICL 2156-7085 10.1364/BOE.10.002795 31259052 PMC6583357

[r4] Vidal-Rosas E. E. et al., “Evaluating a new generation of wearable high-density diffuse optical tomography technology via retinotopic mapping of the adult visual cortex,” Neurophotonics 8(2), 025002 (2021). 10.1117/1.NPh.8.2.025002 33842667 PMC8033536

[5] Yücel M. A. et al., “Best practices for fNIRS publications,” Neurophotonics 8(1), 012101 (2021). 10.1117/1.NPh.8.1.012101 33442557 PMC7793571

[6] Lange F. Tachtsidis I., “Clinical brain monitoring with time domain NIRS: a review and future perspectives,” Appl. Sci. 9(8), 1612 (2019). 10.3390/app9081612

[7] Boyden E. S. et al., “Millisecond-timescale, genetically targeted optical control of neural activity,” Nat. Neurosci. 8(9), 1263–1268 (2005). NANEFN 1097-6256 10.1038/nn1525 16116447

[r8] Deisseroth K., “Optogenetics: 10 years of microbial opsins in neuroscience,” Nat. Neurosci. 18(9), 1213–1225 (2015). NANEFN 1097-6256 10.1038/nn.4091 26308982 PMC4790845

[9] Wirdatmadja S. et al., “Analysis of light propagation on physiological properties of neurons for nanoscale optogenetics,” IEEE Trans. Neural Syst. Rehabil. Eng. 27(2), 108–117 (2019). 10.1109/TNSRE.2019.2891271 30624220

[10] Liu Y. et al., “OptogenSIM: a 3D Monte Carlo simulation platform for light delivery design in optogenetics,” Biomed. Opt. Express 6(12), 4859–4870 (2015). BOEICL 2156-7085 10.1364/BOE.6.004859 26713200 PMC4679260

[11] Salehpour F. et al., “Penetration profiles of visible and near-infrared lasers and light-emitting diode light through the head tissues in animal and human species: a review of literature,” Photobiomodul. Photomed. Laser Surg. 37(10), 581–595 (2019). 10.1089/photob.2019.4676 31553265

[r12] Lin H. et al., “Transcranial photobiomodulation for brain diseases: review of animal and human studies including mechanisms and emerging trends,” Neurophotonics 11(1), 010601 (2024). 10.1117/1.NPh.11.1.010601 38317779 PMC10840571

[13] Shanshool A. S. et al., “Advances in the transport of laser radiation to the brain with optical clearing: from simulation to reality,” Prog. Quantum Electron. 94, 100506 (2024). PQUEAH 0079-6727 10.1016/j.pquantelec.2024.100506

[14] Jeurissen B. et al., “Diffusion MRI fiber tractography of the brain,” NMR Biomed. 32(4), e3785 (2019). NMRBEF 0952-3480 10.1002/nbm.3785 28945294

[15] Svaasand L. O. Ellingsen R., “Optical properties of human brain,” Photochem. Photobiol. 38(3), 293–299 (1983). PHCBAP 0031-8655 10.1111/j.1751-1097.1983.tb02674.x 6634962

[r16] Taddeucci A. et al., “Optical properties of brain tissue,” J. Biomed. Opt. 1(1), 117–123 (1996). JBOPFO 1083-3668 10.1117/12.227816 23014652

[r17] Bevilacqua F. et al., “In vivo local determination of tissue optical properties: applications to human brain,” Appl. Opt. 38(22), 4939–4950 (1999). APOPAI 0003-6935 10.1364/AO.38.004939 18323984

[r18] Yaroslavsky A. et al., “Optical properties of selected native and coagulated human brain tissues in vitro in the visible and near infrared spectral range,” Phys. Med. Biol. 47(12), 2059 (2002). 10.1088/0031-9155/47/12/305 12118601

[r19] Gebhart S. Lin W. Mahadevan-Jansen A., “In vitro determination of normal and neoplastic human brain tissue optical properties using inverse adding-doubling,” Phys. Med. Biol. 51(8), 2011 (2006). 10.1088/0031-9155/51/8/004 16585842

[20] Jacques S. L., “Optical properties of biological tissues: a review,” Phys. Med. Biol. 58(11), R37 (2013). 10.1088/0031-9155/58/11/R37 23666068

[21] Anand S. et al., “Effects of formalin fixation on tissue optical properties of in-vitro brain samples,” Proc. SPIE 9321, 93210Z (2015). PSISDG 0277-786X 10.1117/12.2076961

[22] Cletus B. et al., “Temperature-dependent optical properties of intralipid® measured with frequency-domain photon-migration spectroscopy,” J. Biomed. Opt. 15(1), 017003 (2010). JBOPFO 1083-3668 10.1117/1.3290820 20210477

[23] Iorizzo T. W. et al., “Temperature induced changes in the optical properties of skin in vivo,” Sci Rep 11(1), 754 (2021). 10.1038/s41598-020-80254-9 33436982 PMC7803738

[24] Hebeda K. M. et al., “Light propagation in the brain depends on nerve fiber orientation,” Neurosurgery 35(4), 720–724 (1994). NEQUEB 10.1227/00006123-199410000-00019 7808616

[25] Heiskala J. et al., “Modeling anisotropic light propagation in a realistic model of the human head,” Appl. Opt. 44(11), 2049–2057 (2005). APOPAI 0003-6935 10.1364/AO.44.002049 15835354

[26] Heiskala J. et al., “Significance of tissue anisotropy in optical tomography of the infant brain,” Appl. Opt. 46(10), 1633–1640 (2007). APOPAI 0003-6935 10.1364/AO.46.001633 17356605

[27] DePaoli D. et al., “Anisotropic light scattering from myelinated axons in the spinal cord,” Neurophotonics 7(1), 015011 (2020). 10.1117/1.NPh.7.1.015011 32206678 PMC7063473

[28] Menzel M. et al., “Toward a high-resolution reconstruction of 3D nerve fiber architectures and crossings in the brain using light scattering measurements and finite-difference time-domain simulations,” Phys. Rev. X 10(2), 021002 (2020). PRXHAE 2160-3308 10.1103/PhysRevX.10.021002

[29] Menzel M. et al., “Scatterometry measurements with scattered light imaging enable new insights into the nerve fiber architecture of the brain,” Front. Neuroanat. 15, 767223 (2021). 10.3389/fnana.2021.767223 34912194 PMC8667079

[30] Kumar S. et al., “Possible existence of optical communication channels in the brain,” Sci. Rep. 6(1), 36508 (2016). 10.1038/srep36508 27819310 PMC5098150

[31] Liu G. et al., “Myelin sheath as a dielectric waveguide for signal propagation in the mid-infrared to terahertz spectral range,” Adv. Funct. Mater. 29(7), 1807862 (2019). AFMDC6 1616-301X 10.1002/adfm.201807862

[32] Pini E. et al., “Diffusion of light in structurally anisotropic media with uniaxial symmetry,” Phys. Rev. Res. 6(2), 023051 (2024). 10.1103/PhysRevResearch.6.023051

[33] Alerstam E., “Anisotropic diffusive transport: connecting microscopic scattering and macroscopic transport properties,” Phys. Rev. E 89(6), 063202 (2014). PLEEE8 1539-3755 10.1103/PhysRevE.89.063202 25019904

[34] Pini E. et al., “Time-resolved light transport in structurally anisotropic media,” Proc. SPIE 12856, 1285606 (2024). PSISDG 0277-786X 10.1117/12.3002766

[35] Pini E. et al, “Experimental determination of effective light transport properties in fully anisotropic media,” Adv. Photonics Nexus 3(5), 056017 (2024). 10.1117/1.APN.3.5.056017

[36] Basser P. J. Pierpaoli C., “Microstructural and physiological features of tissues elucidated by quantitative-diffusion-tensor MRI,” J. Magn. Reson. 213(2), 560–570 (2011). 10.1016/j.jmr.2011.09.022 22152371

[37] Budde M. D. Frank J. A., “Examining brain microstructure using structure tensor analysis of histological sections,” Neuroimage 63(1), 1–10 (2012). NEIMEF 1053-8119 10.1016/j.neuroimage.2012.06.042 22759994

[38] Pattelli L. et al., “Spatio-temporal visualization of light transport in complex photonic structures,” Light Sci. Appl. 5(5), e16090 (2016). 10.1038/lsa.2016.90 30167167 PMC6059935

[39] Costantini I. et al., “A cellular resolution atlas of Broca’s area,” Sci. Adv. 9(41), eadg3844 (2023). 10.1126/sciadv.adg3844 37824623 PMC10569704

[40] Sorelli M. et al., “Myelinated fiber labeling and orientation mapping of the human brain with light-sheet fluorescence microscopy,” bioRxiv (2025). 10.1016/j.neuroimage.2025.121581 PMC12706767 41270845

[41] Sorelli M. et al., “Fiber enhancement and 3D orientation analysis in label-free two-photon fluorescence microscopy,” Sci. Rep. 13(1), 4160 (2023). 10.1038/s41598-023-30953-w 36914673 PMC10011555

[42] Costantini I. et al., “Large-scale, cell-resolution volumetric mapping allows layer-specific investigation of human brain cytoarchitecture,” Biomed. Opt. Express 12(6), 3684–3699 (2021). BOEICL 2156-7085 10.1364/BOE.415555 34221688 PMC8221968

[43] Truong T.-K. Guidon A. Song A. W., “Cortical depth dependence of the diffusion anisotropy in the human cortical gray matter in vivo,” PLoS One 9(3), e91424 (2014). POLNCL 1932-6203 10.1371/journal.pone.0091424 24608869 PMC3946789

[44] Cooley S. A. et al., “Posterior brain white matter abnormalities in older adults with probable mild cognitive impairment,” J. Clin. Exp. Neuropsychol. 37(1), 61–69 (2015). JCENE8 10.1080/13803395.2014.985636 25523313 PMC4355053

[45] Naglič P. et al., “PyXOpto: an open-source python library with utilities for fast light propagation modeling in turbid media,” in Eur. Conf. Biomed. Opt., Optica Publishing Group, p. EM3C–2 (2021).

[46] Naglič P. et al., “Massively parallel Monte Carlo simulations of light propagation in anisotropic scattering media by open-source PyXOpto engine,” Proc. SPIE PC12856, PC1285608 (2024). PSISDG 0277-786X 10.1117/12.3001838

[47] Khan R. et al., “Refractive index of biological tissues: review, measurement techniques, and applications,” Photodiagn. Photodyn. Ther. 33, 102192 (2021). 10.1016/j.pdpdt.2021.102192 33508501

[48] Svensson T. et al., “Exploiting breakdown of the similarity relation for diffuse light transport: simultaneous retrieval of scattering anisotropy and diffusion constant,” Opt. Lett. 38(4), 437–439 (2013). OPLEDP 0146-9592 10.1364/OL.38.000437 23455094

[49] Shariati B. et al., “Method for tissue clearing: temporal tissue optical clearing,” Biomed. Opt. Express 13(8), 4222–4235 (2022). BOEICL 2156-7085 10.1364/BOE.461115 36032583 PMC9408250

[50] Lewis A. V. Fang Q., “Revisiting equivalent optical properties for cerebrospinal fluid to improve diffusion-based modeling accuracy in the brain,” Neurophotonics 12(1), 015009 (2025). 10.1117/1.NPh.12.1.015009 39957838 PMC11828630

[51] Okada E. Delpy D. T., “Near-infrared light propagation in an adult head model. I. Modeling of low-level scattering in the cerebrospinal fluid layer,” Appl. Opt. 42(16), 2906–2914 (2003). APOPAI 0003-6935 10.1364/AO.42.002906 12790439

[52] Galiakhmetova D. et al., “Ultra-short laser pulses propagation through mouse head tissues: experimental and computational study,” IEEE J. Sel. Top. Quantum Electron. 29(4: Biophotonics), 1–11 (2022). IJSQEN 1077-260X 10.1109/JSTQE.2022.3214788

[53] Nickell S. et al., “Anisotropy of light propagation in human skin,” Phys. Med. Biol. 45(10), 2873 (2000). 10.1088/0031-9155/45/10/310 11049177

[54] Kienle A. Forster F. Hibst R., “Anisotropy of light propagation in biological tissue,” Opt. Lett. 29(22), 2617–2619 (2004). OPLEDP 0146-9592 10.1364/OL.29.002617 15552663

[55] Binzoni T. et al., “Anisotropic photon migration in human skeletal muscle,” Phys. Med. Biol. 51(5), N79 (2006). 10.1088/0031-9155/51/5/N01 16481676

[56] Simon E. Krauter P. Kienle A., “Time-resolved measurements of the optical properties of fibrous media using the anisotropic diffusion equation,” J. Biomed. Opt. 19(7), 075006 (2014). JBOPFO 1083-3668 10.1117/1.JBO.19.7.075006 25055055

[57] Nazarian A. A., “Structure of tendon causes highly anisotropic optical properties and transport,” Master’s thesis, Northeastern University (2024).

[58] Soltaninezhad M. et al., “Optical anisotropy measurement in normal and cancerous tissues: backscattering technique,” Biomed. Opt. Express 11(6), 2996–3008 (2020). BOEICL 2156-7085 10.1364/BOE.393079 32637237 PMC7316004

[59] Radwan A. M. et al., “An atlas of white matter anatomy, its variability, and reproducibility based on constrained spherical deconvolution of diffusion MRI,” NeuroImage 254, 119029 (2022). NEIMEF 1053-8119 10.1016/j.neuroimage.2022.119029 35231632 PMC10265547

[60] Sassaroli A. Blaney G. Fantini S., “Dual-slope method for enhanced depth sensitivity in diffuse optical spectroscopy,” J. Opt. Soc. Am. A 36(10), 1743–1761 (2019). JOAOD6 0740-3232 10.1364/JOSAA.36.001743 PMC7160974 31674440

[61] Blaney G. et al., “Dual-slope imaging of cerebral hemodynamics with frequency-domain near-infrared spectroscopy,” Neurophotonics 10(1), 013508 (2023). 10.1117/1.NPh.10.1.013508 36601543 PMC9807277

[62] Zhang B. et al., “Effect of incidence sites on light distribution at different wavelengths during transcranial photobiomodulation,” Acad. Radiol. in press (2025). 10.1016/j.acra.2025.04.076 40410107

[63] Radford J. et al., “Photon transport through the entire adult human head,” Neurophotonics 12(2), 025014 (2025). 10.1117/1.NPh.12.2.025014 40438285 PMC12117216

[64] Li Z. et al., “A statistical skull geometry model for children 0-3 years old,” PLoS One 10(5), e0127322 (2015). POLNCL 1932-6203 10.1371/journal.pone.0127322 25992998 PMC4436309

[65] Amendola C. et al., “Accuracy of homogeneous models for photon diffusion in estimating neonatal cerebral hemodynamics by TD-NIRS,” Biomed. Opt. Express 12(4), 1905–1921 (2021). BOEICL 2156-7085 10.1364/BOE.417357 33996206 PMC8086468

[66] Buyanova I. S. Arsalidou M., “Cerebral white matter myelination and relations to age, gender, and cognition: a selective review,” Front. Hum. Neurosci. 15, 662031 (2021). 10.3389/fnhum.2021.662031 34295229 PMC8290169

[67] Russomanno E. et al., “Effects of different optical properties of head tissues on near-infrared spectroscopy using Monte Carlo simulations,” in Oxygen Transp. to Tissue XLIII, pp. 39–43 (2022). 10.1007/978-3-031-14190-4_7 36527611

[68] Costantini I. et al., “Autofluorescence enhancement for label-free imaging of myelinated fibers in mammalian brains,” Sci. Rep. 11(1), 8038 (2021). 10.1038/s41598-021-86092-7 33850168 PMC8044204

[69] Suzuki H. et al., “Hemodynamic measurements of the human adult head in transmittance mode by near-infrared time-resolved spectroscopy,” in Oxygen Transp. Tissue XXXVII, Springer, pp. 399–406 (2016). 10.1007/978-1-4939-3023-4_50 26782238

